# The Spectrum of Treatment Modalities for Gastroesophageal Reflux Disease (GERD): A Narrative Review

**DOI:** 10.7759/cureus.32619

**Published:** 2022-12-17

**Authors:** Namrata R Velagala, Vivek R Velagala, Yashwant Lamture

**Affiliations:** 1 Medicine, Jawaharlal Nehru Medical College, Datta Meghe Institute of Medical Sciences, Wardha, IND; 2 Surgery, Jawaharlal Nehru Medical College, Datta Meghe Institute of Medical Sciences, Wardha, IND

**Keywords:** gerd, gerd pathophysiology, acid reflux, laparoscopic treatment, gastroesophageal reflux disease (gerd), proton-pump inhibitors (ppi)

## Abstract

Gastroesophageal reflux disease (GERD) is a prevalent gastrointestinal disease that is encountered and treated by physicians all over the world. It is a chronic and non-progressive condition. Symptoms can range from mild heartburn to chronic, recurrent, and severe symptoms like constant acid regurgitation, laryngitis, bad breath, otitis media, and severe heartburn, which can be debilitating to the patient. Hence, the administration of appropriate therapy according to the patient's severity of symptoms is imperative, more so because over-the-counter drugs like antacids are very common to treat GERD. Often, in some instances, mere changes in lifestyle prove highly effective in reversing GERD symptomatology. Depending on the severity, response to treatment, and presence or absence of complications, treatment with medical or surgical modalities can be decided. It has now been found that although the gold standard in medical therapy for GERD has been proton pump inhibitors (PPIs), there has been increasing research about their side effects and recurrence after treatment. Hence, newer anti-GERD drugs have been under trial, which has been discussed in detail in the review. The use of surgical fundoplications has drastically decreased and is being widely replaced by incisionless laparoscopic fundoplications and newer endoluminal techniques such as the LINX device. This review aims to compile the vast spectrum of treatment modalities for GERD, ranging from more contemporary diagnostic methods, lifestyle modifications, medical therapy, and surgical and endoluminal techniques, with a particular focus on newer directions.

## Introduction and background

As per the Montreal definition, the reflux of the contents of the stomach back into the esophagus is called gastroesophageal reflux disease (GERD), and it is usually associated with a variety of complex symptoms and complications. [1]. GERD occurs mainly due to the reflux of gastric contents into the esophagus, causing esophageal injury. It is a chronic condition that is generally non-progressive [2]. The causes behind the occurrence of this syndrome complex are decreased pressure in the lower esophageal sphincter, a decrease in the length of the intrabdominal esophagus, hiatal hernia, an increase in the intrabdominal pressure, and disturbances in the esophageal clearance [3]. The most common cause is transient lower esophageal sphincter relaxations (TSLERS), which inhibit the lower esophageal sphincter tone for short durations, irrespective of swallowing. Although they are physiological, in GERD, their frequency increases so much that it causes increased exposure to the refluxate [4]. GERD can present as erosive esophagitis, non-erosive esophagitis, or Barrett's esophagus, the latter being a premalignant condition [5]. GERD should be differentiated from GER (Gastro-esophageal reflux), which is a physiological passage of gastric contents into the esophagus that occurs several times throughout the day in healthy individuals and is not associated with exasperating symptoms and/or complications, contrary to GERD. Inferring a difference between the two is important to avoid the administration of acid-suppressing drugs when they are not required [6]. Non-erosive reflux disease (NERD) is used for patients experiencing reflux symptoms but showing no evidence of mucosal breaks [7]. In the other two phenotypes, on endoscopy, there is an apparent injury to the mucosal layer of the esophagus. One prominent pathological hallmark of NERD, detected on electron or light microscopy, is the presence of dilated intracellular spaces in the squamous epithelium. On 24-hour pH monitoring, it was found that only 50% of NERD patients have pathological esophageal exposure. Such patients are considered to have esophageal hypersensitivity [8]. The presence of a hiatal hernia has also been shown to worsen lower esophageal sphincter action, resulting in increased acid exposure [9]. 

Prevalence

GERD is prevalent all over the world, with the burden of the disease showing an increasing trend. Estimates show that the prevalence of GERD is about 18%-27% in North America, 23% in South America, 8%-25% in Europe, 2.5%-8% in East Asia, 8-33% in the Middle East, and 11.6% in Australia [10]. It is estimated that about six billion dollars are spent annually in the United States on anti-GERD medication [11]. The last decade has seen a drastic change in management trends for GERD. It has been observed that alternatives, due to increased side effects, are replacing widely used proton pump inhibitors (PPI). There has also been a decrease in the application of surgical fundoplications, which are being replaced by less invasive endoluminal surgeries. Other newer modalities are also being employed along with those under trial for future use. These have been discussed in this review [12].

Risk factors for GERD

GERD is a prevalent gastrointestinal disorder that can develop due to many factors. These risk factors include both modifiable and non-modifiable components. The non-modifiable ones are age, sex, and genetics. Modifiable ones are lifestyle, eating habits, and increased body mass index (BMI). Smoking, eating spicy, fatty, or fried food, drinking alcohol, engaging in rigorous physical activity after meals or less rigorous physical activity overall, and consuming chocolates, carbonated beverages, coffee, and tea are all lifestyle components. Even grapes, tomatoes, and preservatives have also been listed as risk factors for developing GERD. Poor eating habits, such as eating large meals at once, eating right before going to bed, and irregular meal timings, all contribute to the development and aggravation of GERD symptoms [13]. Consumption of certain drugs also poses a risk to the emergence of GERD, namely non-steroid anti-inflammatory drugs (NSAIDs), hormone replacement therapy, anti-depressants, benzodiazepines, and theophylline [14].

Common manifestations

**Figure 1:**
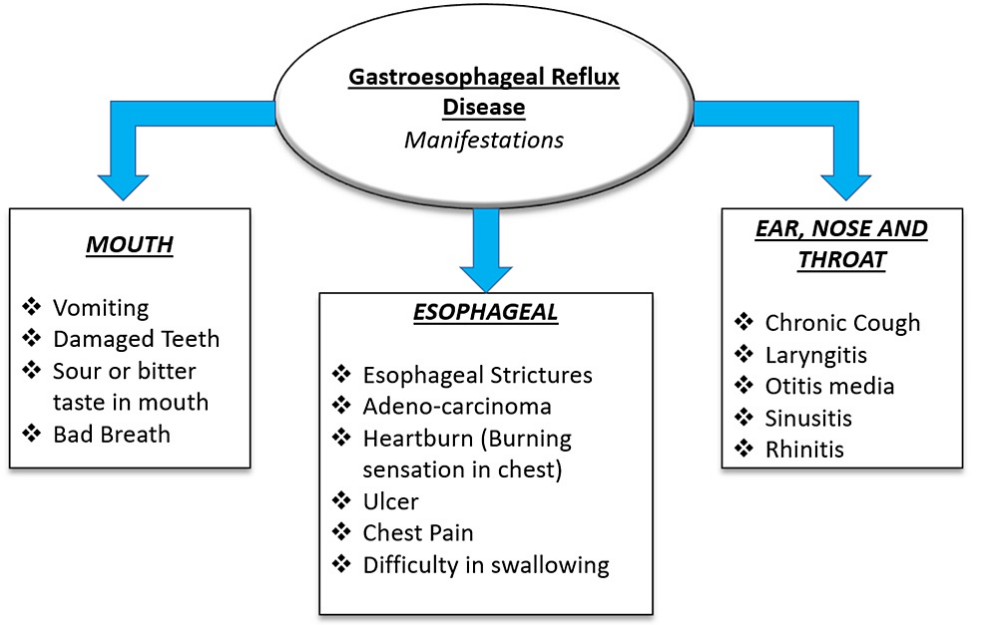
Manifestations of gastroesophageal reflux disease The image has been created by the authors.

The most common symptoms of GERD are heartburn, chest pain, and regurgitation of acid. Other severe manifestations include esophageal strictures, ulcers, and adenocarcinoma [15]. GERD also significantly affects the ear, nose, and throat (ENT), resulting in various manifestations, including chronic cough, rhinitis, sinusitis, otitis media, and laryngitis [16]. The common manifestations are shown in Figure 1.

Diagnosis of GERD

The diagnosis of GERD made only through history is deemed insufficient; further investigations are required to complete a definite diagnosis of GERD. The response to empiric PPI therapy is noted in the patients and based on the results, the diagnosis proceeds. No response indicates the need for further testing to be done, which can only be conducted if the patient is off the PPI treatment for at least 2 weeks [17]. These tests include the Bravo pH capsule, which is considered very promising and safer. Endoscopy is a commonly employed diagnostic technique to check for complications, but it has low sensitivity. Other newly developed diagnostic modalities are multichannel intraluminal impedance-pH monitoring, a non-invasive GERD marker called Gastrin-17, the post-reflux swallowing peristaltic wave index (PSPWI), and the mean nocturnal basal impedance (MNBI) [18-20].

## Review

Lifestyle modifications

In managing GERD, one of the most important interventions is lifestyle modification, which is usually ignored by physicians and not adhered to by patients. Avoiding foods that relax the lower esophageal sphincter, like chocolate, spicy food, caffeine, tobacco, and alcohol, is recommended [12]. The relationship between diet and GERD has been studied in order to provide patients with a more practical approach [21]. Consumption of certain beverages should be avoided for managing GERD, like acidic beverages, which are known to worsen GERD and precipitate its symptoms [22]. Carbonated drinks also alter intrabdominal pressure and increase transient lower esophageal sphincter relaxation frequency [23]. Besides beverages, patients are also counseled to reduce their intake of certain spices. One of them is mint, which has been known to act as a trigger in a small group of patients [24]. Intake of spicy food also acts as an irritant to the esophageal mucosa [25]. Manipulation of the macronutrients is very commonly employed nowadays as a dietary intervention. Macronutrients include fats, carbohydrates, and proteins. The correlation between the ingestion of fats and GERD needs to be researched more, as studies show contradicting results. A study of 12 healthy individuals compared a low-calorie meal to a high-calorie one. Still, it was found not to affect lower esophageal sphincter pressures, whereas another study showed different results, indicating increased acid reflux exposure due to the ingestion of fatty meals [26-28]. In addition to avoiding certain beverages, manipulation of macronutrients, eating habits, and patterns are also implicated in the development and aggravation of GERD symptoms. Having dinner on time is highly recommended, as it maintains a sustained release of pH at night, preventing reflux episodes in the supine position during sleep [21]. A recent study on Albanian patients found that a Mediterranean diet, which includes a lot of fruits, vegetables, and whole grains, is associated with a lower incidence of GERD-related symptoms [29]. In 2000, a survey was conducted to infer the relation between BMI and the severity of symptoms in GERD. Around 10,545 women were included in the study, which was conducted with the help of a questionnaire. It was found that even a slight increase in BMI could exacerbate the symptoms of GERD. Women who had reduced their BMI by around 3.5 units were found to have a drastic reduction in the symptoms of GERD by approximately 40% [30]. Hence, weight reduction is a substantial lifestyle modification that a patient with GERD should inculcate. Another review focusing on the correlation between sleep pattern and sleeping position with GERD states that sleep affects gastric emptying and esophageal peristalsis, and secretions are also reduced during sleep. Hence, shorter sleep duration and sleep disturbance lead to gastric reflux symptoms. It was found that chronic sleep deprivation can cause esophageal hyperplasia due to the constant reflux of acid. Sleeping in the right decubitus position and having meals three hours before sleeping is a fundamental and practical lifestyle intervention for improving GERD symptoms [31].

Medical management

**Figure 2:**
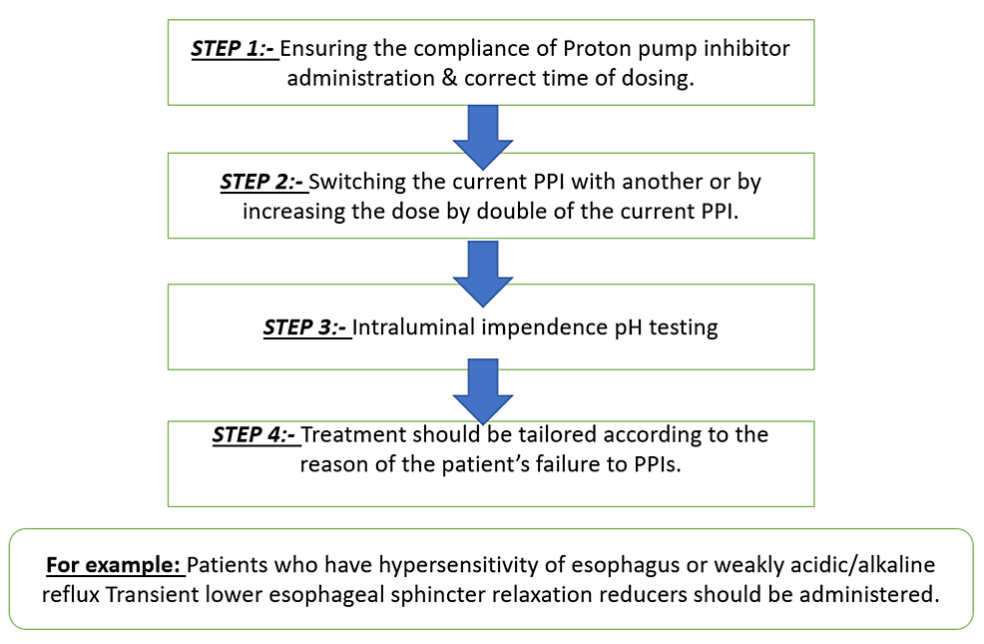
Management of refractory gastroesophageal reflux disease PPIs: proton pump inhibitors The image has been created by the authors.

Despite adopting various lifestyle modifications, a specific section of patients continues to experience exasperating reflux symptoms, and medical therapy is recommended for them. Medical therapy comprises histamine H2 receptor antagonists (H2RAs), PPIs, TLESR reducers, and prokinetics [12]. PPIs and H2RAs are the most commonly used drugs [32]. Prokinetics are not as widely used as the former two PPIs and H2RAs, which act by reducing the release of acid from the stomach. At the same time, prokinetics increase the lower esophageal (LES) tone, accelerating gastric emptying and reducing acid exposure to the esophagus. Proton pump inhibitors have also been shown to be more effective than H2RAs in treating patients who are receiving empirical treatment or show no signs of esophageal damage on upper gastrointestinal endoscopy. Patients undergoing empirical treatment are the ones who experience reflux symptoms but have not undergone any diagnostic tests for GERD, like an upper gastrointestinal endoscopy [33]. The initial full-dose medical treatment consists of a 20 mg omeprazole tablet taken once daily [34]. However, proton pump inhibitors, the most widely prescribed anti-GERD drug, also have many side effects and unmet needs. Long-term use, or overuse, of PPIs has been found to lead to secondary hypergastrinemia, increased susceptibility to infections, and altered absorption of micronutrients. It is also known to cause rebound acid hypersecretion on cessation of long-term medication [35]. Long-term use of PPIs also increases the risk of benign paroxysmal positional vertigo (BPPV) in adult patients [36]. Although PPIs have proven to be the gold standard for the empirical treatment of GERD, many patients remain symptomatic even after taking standard PPI therapy. Hence, newer drugs in the pharmacological arena of treatment are being researched even more to meet the unmet needs of the current line of treatment. These drug classes comprise gamma-aminobutyric acid type-B (GABA-B) receptor agonists like lesogaberan, arbaclofen placarbil, metabotropic glutamate receptor-5 (mGLuR5) antagonists, potassium-competitive acid blockers (P-CABs), cholecystokinin antagonists, mosapride, and rikkunshito as add-on drugs to PPIs [37]. In the case of refractory GERD, which means not responding to empirical treatment with PPIs, there can be various causes like psychological morbidities, weakly acidic or alkaline reflux, and hypersensitivity of the esophagus [38]. The management of refractory GERD is shown in Figure 2.

Surgical management

Several surgical strategies are present for the treatment of GERD. However, a survey found that there has been a recent decline in the utilization of surgical fundoplications in the United States, especially in the years 2009-2013 [39]. Although surgical fundoplication, which is considered the gold standard in such cases, may benefit patients whose symptoms are unmanageable with PPI therapy, post-fundoplication, the majority of patients have expressed satisfaction. However, mild bloating, dysphagia and the resumption of PPI therapy post-surgery are also common [40]. Another study was conducted in order to compare the mechanical efficiency of the three types of fundoplications, Dor and Toupet, which are partial fundoplications, and the complete Nissen fundoplication, and it was found that the esophagogastric junction (EGJ) was more distensible post-Dor fundoplication, whereas there was no significant difference in distensibility between Nissen and Toupet fundoplication. Despite this, failure point measurements revealed that Nissen was more effective than Toupet, and Toupet was more effective than Dor [41]. Laparoscopic Nissen's fundoplication has also proven to be a very effective treatment modality, with the advantages of being minimally invasive and able to repair any hiatal defect present. Also, the necessity of long-term medication comes to an end. As Nissen's fundoplication requires immense precision and is minimally invasive, it demands modern equipment and a highly trained and experienced surgeon [42]. It has also been observed that anterior partial fundoplication (AF) has fewer mechanical side effects than Nissen's complete fundoplication (NF). A randomized control trial was conducted to compare the long-term results of both types of fundoplication. About 100 patients who underwent laparoscopic AF and laparoscopic NF were enrolled in the study, and postoperative complaints like flatulence and dyspepsia were taken into account. Post-surgery, both AF and NF proved to show reasonable control of reflux-related symptoms in patients. However, a significantly lower number of patients in the AF group complained of flatulence after the surgery [43].

Endoluminal techniques and new directions

The advent of newer endoluminal techniques has made incision-less treatment of GERD possible. Various new modalities include injectable polymers to tighten the lower esophageal sphincter, anti-reflux mucosectomy, radio-frequency ablation (RFA), endoscopic suturing devices, electrical stimulation of the lower esophageal sphincter, and Collis gastroplasty for the management of hiatal hernia complicated by GERD [44,45]. A new endoscopic treatment modality is transoral incisionless fundoplication (TIF), and an explorative study was conducted to find the effect of TIF on two reflux mechanisms, TLESRs and esophagogastric junction distensibility (EGJ). Transoral incisionless fundoplication was found to reduce the number of TLESRs postprandially as well as esophagogastric junction distensibility. It was also noted that the TIF effect was specific to liquid reflux, allowing gastric air to pass [46]. Other endoluminal techniques involve injectable polymers, which increase the competency of the lower esophageal sphincter (LES). These injectables are bulking agents: ethylene vinyl alcohol copolymers, popularly called Enteryx and Durasphere. Durasphere's efficacy and safety were investigated in a study of 10 GERD patients with confirmed cases by pH monitoring and mild-moderate esophagitis [47]. It was conferred that Durasphere was a favorable injectable agent with no notable side effects. In multiple clinical trials, it was found that Enteryx effectively reduced proton pump usage in about 84% of patients in one year and by about 72% in two years [47,48]. Recently, a newer technique called magnetic sphincter augmentation (MSA), also known as the LINX device, has been used. It is an implantable ring with magnetic beads put around the esophagus to restore the adequate length of the intrabdominal esophagus. According to the literature available, it is observed that the LINX device is not only as effective as Nissen's fundoplication but is a safe treatment modality. The most common complication post-LINX device is usually dysphagia, which resolves independently. Device erosion has rarely been reported to occur. But the device can be easily and safely removed in the very few reported cases of erosion [49].

## Conclusions

GERD is a common condition that can be easily managed by inculcating lifestyle modifications and empirical medical therapy. Careful assessment before diagnosing GERD is crucial to rule out complications like strictures, malignancy, and Barrett's esophagus. The diagnosis is usually made by 24-hour pH monitoring and upper GI endoscopy. Still, newer techniques like the Bravo pH capsules, multichannel intraluminal impedance-pH monitoring, and non-invasive procedures such as the detection of GERD markers like "Gastrin-17" have come into practice. Lifestyle modifications, often neglected by both patients and physicians, have been shown to affect the prognosis of the disease far more than presumed. Hence, if mindfully followed, it can show a drastic improvement in symptomatology. In medical therapy, proton pump inhibitors are the gold standard. However, their side effects and recurrence after treatment have recently come to light. In such cases, optimization of the dosing or switching to other groups of drugs, such as H2RAs and prokinetic agents, should be considered. Various newer drugs like GABA receptor agonists, cholecystokinin antagonists, and P-CABs are also being researched and are currently under trial. Surgical fundoplications for refractory GERD are still performed but have drastically decreased, perhaps due to the advent of incisionless laparoscopic techniques. Laparoscopic Nissen's fundoplication has become quite popular and common in practice as it has the advantage of being minimally invasive and has also been known to correct the hiatal defects present. Newer endoluminal techniques like the LINX device, radio-frequency ablations, injectable polymers, and transoral incisionless fundoplications have proven to be quite effective. Also, they serve as an excellent alternative to long-term medical treatment and surgeries.
